# Fever enhances host bacterial defence while limiting mitochondrial damage

**DOI:** 10.21203/rs.3.rs-8724408/v1

**Published:** 2026-02-06

**Authors:** Yonghan Wu, Elijah Rowe, Albert Siryaporn, Steven P. Gross

**Affiliations:** 1 Department of Physics and Astronomy, University of California Irvine, Irvine, California, USA.; 2 School of Biological Sciences, University of California Irvine, Irvine, California, USA.; 3 Department of Molecular Biology and Biochemistry, University of California Irvine, Irvine, California, USA.; 4 Department of Developmental and Cell Biology, University of California Irvine, Irvine, California, USA.

## Abstract

The fever response is triggered during infection and inflammation, but the importance of this response for the function of antimicrobial peptides (AMPs) remains unknown. We discovered that the activity of the human AMP LL-37 is temperature-dependent and were curious as to why. Its temperature-dependent activity is not an enzymatic accident, as AMPs from other animals with different body temperatures have distinct temperature sensitivities. We find that in general, AMP temperature sensitivities are tuned to animals’ body temperatures, and that for animals that can induce fever or raise their body temperature, this is used to increase the AMP’s antibacterial efficacy. This effect reflects a careful balance between optimizing antibacterial killing while minimizing mitochondrial damage. In contrast, cold-blooded animals that are unable to raise their body temperature use a different strategy to avoid mitochondrial damage. Together, this study suggests that fever is beneficial for antimicrobial defence by raising AMP activity towards bacteria while reducing mitochondrial damage when pathogens are absent.

Animals have evolved distinct thermoregulatory strategies. Mammals maintain their body temperatures around 37°C, whereas amphibians and reptiles allow the environment to adjust their body to temperature typically lower than that of mammals. Following infection, the body temperature of mammals rises as part of the fever response. Snakes can actively raise their body temperature by basking under sunlight^1^, while most other cold-blooded animals lack this ability. Both the innate and adaptive immune systems are stimulated by elevated temperature^2^. However, how elevated temperature leads to improved clearance of pathogens in hosts is not understood at the molecular level^3^. Bacterial pathogens contain heat shock systems that make them tolerant to elevated temperatures^4^ and some pathogens grow faster at elevated temperatures^5,6^. Furthermore, higher temperatures have significant detrimental effects on host cells, including impairing mitochondrial activity through uncoupling oxidative phosphorylation^7,8^. Given the many potential downsides of elevated temperatures, it is unclear how the strategy could improve host survival.

One important component of innate immunity is antimicrobial peptides (AMPs), which provide a key defence against bacterial infection^9–12^. The effect of temperature on AMPs’ activity is not understood. In the case of bacteriocin AMPs, an increase in temperature decreases the antimicrobial effects of the antimicrobial peptide^13^. For the chickpea AMP legumin, antimicrobial activity is insensitive to temperature over a broad range of 4°C to 37°C^14^. Contributing to the knowledge gap is that most studies on AMPs’ activity are performed at human physiological temperature at 37°C^15,16^ rather than at the host’s baseline body temperature. Since elevated temperatures can destabilize peptide structure, induce structural transitions, or non-specifically alter protein activity^17–20^, elevated temperatures during a fever response would not be expected to produce greater microbial killing.

The human AMP cathelicidin (LL-37) permeabilizes bacterial membranes^21,22^ and is found in neutrophil extracellular traps (NETs) and on lipid droplets, where it colocalizes with histones^23–25^. Histones are best known as condensing DNA in nucleosomes, but have also been found to exhibit antimicrobial activity^26^ through synergistic activity with AMPs^10,27–29^. To better understand this synergy, we more carefully investigated factors that affect AMP activity. Previous synergy studies have shown that a combined treatment of histones and LL-37 induces durable bactericidal pore formation while AMPs alone do not^28^.

We explored the utility of such temperature dependence and suggest that it is not an accident, but rather, reflects a careful balance between optimizing for antibacterial killing while avoiding mitochondrial damage. We observed that the antimicrobial activity of LL-37 is relatively low at room temperature (22°C), elevated at human body temperature (37°C), and highest at febrile temperature (40°C). We then tested AMPs from cold blooded animals such as fish and frogs and found that they all show optimal activity at lower temperatures close to their animals’ living environment. This result suggests that the temperature dependent activity is due to an evolutionarily tuning of the innate immune system rather than faster reaction kinetics. Our data also suggests that it is possible to evolutionarily tune the AMPs’ relative efficacy against bacteria versus mitochondria. At febrile temperature, we observed that mitochondria move away from lipid droplets, protecting themselves from damage by AMPs and histones on lipid droplets. For animals lacking the ability to effect changes in body temperature, a different evolutionary strategy appears to be employed, where AMPs undergo different evolutionary tuning: rather than increasing their temperature-dependent damage, they likely evolved to decrease mitochondrial damage.

## Synergy is amplified by temperature

**Fig. 1 |:**
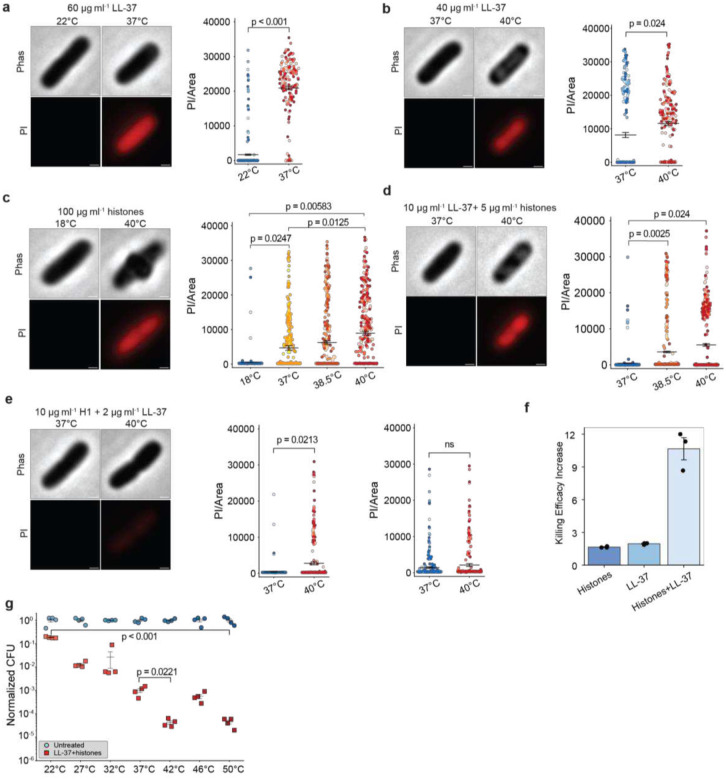
Febrile temperatures increase the antimicrobial activity of human LL-37, histones, and dual treatments. **a**, Representative phase-contrast and fluorescence images of *E. coli* MG1655 treated with 60 μg ml^−1^ LL-37 at room temperature(22°C) and at physiological temperature (37°C). Propidium iodide (PI) fluorescence is quantified and plotted for each cell. **b**, 40 μg ml^−1^ LL-37 treatment at 37°C and 40°C **c**, 100 μg ml^−1^ histones at room temperature, physiological temperature and febrile temperatures. **d**,10 μg ml^−1^ LL-37 and 5 μg ml^−1^ histones at physiological temperature and febrile temperatures **e**, 2 μg ml^−1^ LL-37 and 10 μg ml^−1^ histone H1 (left) and 400 μg ml^−1^ histone H1 alone(right) at body and febrile temperatures. Treatments were performed for 1 h. PI was added after treatment to assess membrane damage **f**, Fold increase in the fraction of cells with PI entry at 40°C compared to 37°C, for treatment with 100 μg ml^−1^ histones, 40 μg ml^−1^ LL-37 and 10 μg ml^−1^ LL-37 and 5 μg ml^−1^ histones. **g**, CFU assays performed at indicated various temperatures for whole histone and LL-37 dual treatment normalized to the untreated control at each temperature. For the PI experiment, three biological replicates were done for each condition and plotted as different color densities. 100 cells were imaged in each replicate. The average fluorescence of each cell is illustrated as a dot in the plot. Black bars indicate the mean and error bars represent standard error of the mean (SEM) from three replicates. Scale bar = 0.5 μm. P-values greater than 0.05 are denoted as non-significant (ns).

We tested histones and LL-37 both alone, and in conjunction with each other, at a range of temperatures (Fig. 1a–d). Activity was measured in two ways: by the ability to permeabilize membranes, as measured by entry of propidium iodide, and by the ability to kill bacteria, assessed by colony-forming unit (CFU) assays. LL-37’s activity was low at sub- physiological body temperatures, increased at body temperature (37°C), and was significantly higher at febrile temperatures (e.g. 38.5°C and 40°C) (Fig. 1a,b). This trend is the same for histones alone (Fig. 1c) and for the LL-37/histones and LL-37/histone H1 dual treatments (Fig. 1d–e). Histone H1 has previously been demonstrated to have antimicrobial activity^30,31^. Notably, there was a massive 11-fold increase in the activity of the synergistic dual treatment at 40°C vs. 37°C (Fig. 1c and Extended Data Fig. 1a and Extended Data Tables 1–2), and at 38.5°C the increase was only slightly less (Extended Data Table 2), at 7-fold. Consistent with this trend, CFU assays showed complete killing at 38.5°C while only partially killing at 37°C (Extended Data Fig. 1b). Temperature rises from room temperature to normal body and febrile temperatures result in significant increases in bacterial killing as assayed by CFUs (Fig. 1g). Thus, the fever response dramatically increases bacterial killing activity. The increase in killing efficacy at febrile temperature is significantly greater for dual treatment than histones or LL-37 alone (Fig. 1f), indicating an advantage of synergy. Since enzymatic activity tends to increase at higher temperatures, we wondered whether this increase was a consequence of reaction kinetics or a more subtle evolutionary tuning mechanism.

## AMP activity matches host temperature

**Fig. 2 |:**
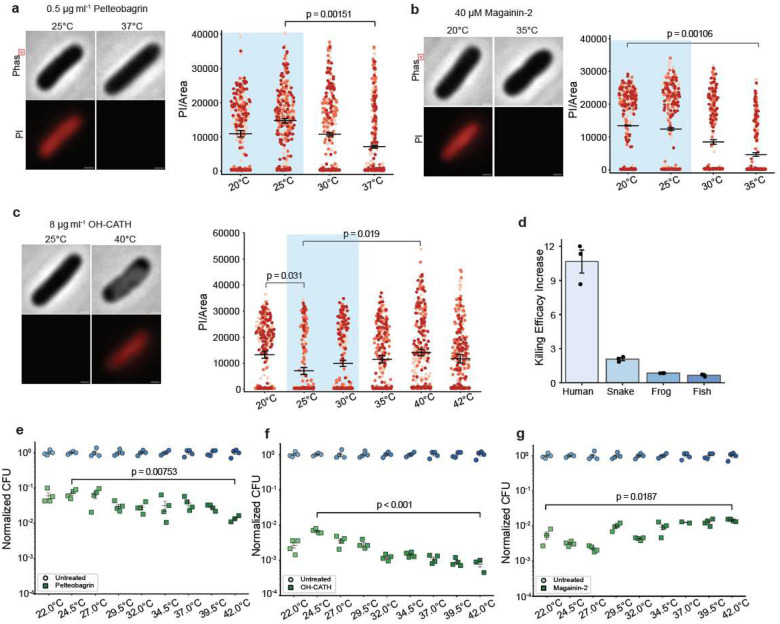
The antimicrobial activity of AMPs is optimized to host body temperature. **a**, Representative images of phase-contrast and fluorescence images of PI entry for *E. coli* treated with Pelteobagrin from catfish. PI fluorescence is quantified and plotted for each cell **b**, Representative images and quantifications for Magainin-2 from the African clawed frog. **c**, Representative images and quantifications for OH-CATH from king cobra. All treatments were conducted on *E. coli* MG1655 for 1 h. **d**, Quantification of fold increase in the number of cells with PI entry at the animals’ optimal living temperature compared to an elevated temperature. 5 μg ml^−1^ histones + 10 μg ml^−1^ LL-37 at 37°C vs. 40°C for human; 8 μg ml^−1^ OH-CATH at 25°C vs. 40°C for snake; 40 μM magainin-2 at 25°C vs. 30°C for frog; 0.5 μg ml^−1^ pelteobagrin at 25°C vs. 30°C for fish. **e–g**, CFU assays following treatment with 10 μg ml^−1^ pelteobagrin (**e**), 2 μg ml^−1^ OH-CATH (**f**) and 2.2 μM Magainin-2 (**g**). Normalization performed identically to fig. 1f. For the PI experiment, three biological replicates were performed for each condition and plotted as different color densities. 100 cells were imaged per replicate. The average fluorescence of each cell is illustrated as a dot in the plot. Black bars indicate the mean and error bars represent SEM from three replicates. Scale bar = 0.5 μm.

To determine whether the increase in LL-37 activity was simply a consequence of temperature enhanced reaction kinetics, we looked at the activity of three other AMPs from species that have lower body temperatures: pelteobagrin (catfish), magainin-2 (frog), and OH-CATH (cobra, snake). We reasoned that if AMP activity was tuned to match body temperature, in these cases it would be maximized at a lower temperature. In contrast, if activity were determined by generic reaction kinetics, it should increase as the temperature increased. In each case, we found that activity matched the typical body temperature ranges of the animals. For instance, the AMP from the yellow catfish was most active around 25°C (Fig. 2a,e and Extended Data Table 3), the typical expected ambient temperature range for this fish^32^, and decreased significantly at 37°C. In contrast the African clawed frog AMP magainin-2 was most active at 20°C (Fig. 2b,g and Extended Data Table 3) and fell monotonically as temperature increased, consistent with their expected temperature range^33^, since these frogs are known to prefer cooler environments. Finally, the snake’s AMP was active over a wide range of temperatures, from 20°C to 40°C (Fig. 2c,f and (Fig. 2a,e and Extended Data Table 3). This is consistent with snake’s exposure to cool (~20°C) temperatures at night, and warmer (30–35°C) temperatures during the day^34^.

We have already noted that LL-37 showed increasing killing activity at elevated body temperatures, such as those reached during fever. Snakes, when infected, often bask on hot rocks in the sun, apparently to raise their body temperature^35,36^. We thus looked carefully at the snake AMP’s activity in a range slightly above their expected body temperatures and found that it was significantly more active (P=0.019), similar to the increased efficacy of the human AMP (P=0.024), though not nearly as extreme as in the human case. We found no such increase in efficacy of the fish or frog AMPs at slightly higher than normal body temperatures (Fig. 2d), consistent with their lack of ability to increase their body temperatures. Since bacterial exposure can happen at any time, one might naively expect that the most powerful antibacterial defence at normal body temperatures would be optimal, yet for humans and snakes, that appears to not be what is occurring. This raised an interesting possibility of some trade-off, and we wondered what it was.

## AMPs and histones damage mitochondria

Both AMPs and histones localize to lipid droplets (LD)^23,37^, which provide fatty acids to mitochondria as a source for fatty acid metabolism^38,39^ in the cytosol. Elevated AMPs concentration during infection might be detrimental because mitochondria have similar membrane physiology to bacteria^40,41^. AMPs permeabilize mitochondrial membranes and trigger apoptosis of mammalian cells^42^. We hypothesize that the temperature dependence of AMPs and histones reflects attempts to moderate this “off target” effect. That is, AMP activity is evolutionarily tuned to be moderate under regular conditions to protect mitochondria, but enhanced during fever when needed to expeditiously kill bacteria.

**Fig. 3 |:**
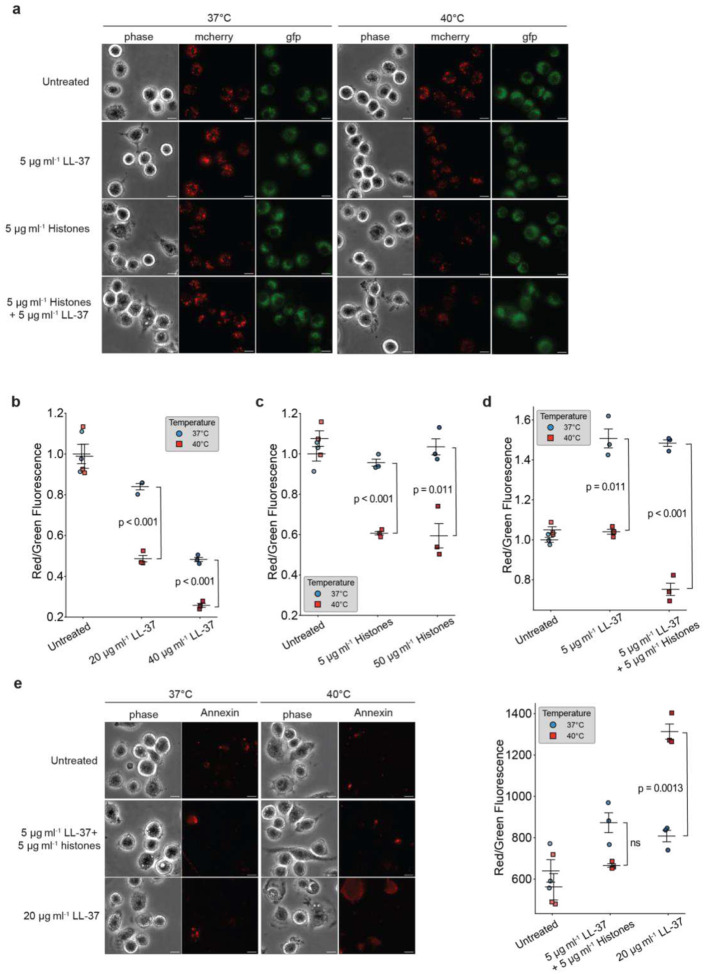
The synergistic antimicrobial activity of histones and LL-37 at febrile temperatures reduces apoptosis. **a**, Representative fluorescence microscopy images of J774.1 macrophages following treatment with 5 μg ml^−1^ histones, 5 μg ml^−1^ LL-37 and the dual treatment. **b-d**, Quantification of JC-1 red/green fluorescence ratio in macrophages treated with LL-37 (**b**), histones (**c**), or the combined treatment (**d**). Decreases in red/green ratios indicate membrane potential changes that are associated with mitochondrial damage. **e**, Representative images and quantifications of Annexin V staining as an indicator of apoptosis. All treatments were performed for 1 hour. 3 biological replicates have been performed for all conditions. Each dot represents one biological replicate. Black bars indicate mean and error bars represent the SEM from three replicates. Scale bar = 10 μm. P-values greater than 0.05 are denoted as non-significant (ns).

In such a model, cells might be insensitive to small or moderate mitochondrial damage at normal body temperatures when pathogens are absent but then tolerant to more significant damage as a worthwhile trade-off when pathogens are present. This would be achieved if AMP and histone activity is temperature dependent and then raising the temperature in response to pathogens—i.e. the fever response. To test this possibility, we cultured normal macrophage J774.1 cells at room temperature and exposed them to a combination of AMPs and histones in the surrounding medium. Because histones and AMPs can cross the eukaryotic cell membrane barrier^43,44^, this external treatment resulted in internal AMPs and histones, allowing them to potentially affect mitochondrial function. We measured mitochondrial health using the JC-1 dye, which shows red fluorescent under high mitochondrial membrane potential and green fluorescence with depolarized mitochondria. Higher red/green ratios are thus associated with mitochondrial health. LL-37 and histones indeed entered cells and caused a dose-dependent decrease in mitochondrial health (Fig. 3a–d). When we raised the temperature to 40°C, mitochondrial damage due to histone, LL-37 and dual treatments was further increased (Fig. 3a–d). Temperature can affect membrane permeability, so the increased mitochondrial damage might be due to increased AMP/histone entry rather than due to the AMP/histone temperature-dependent activity. Since there are free extracellular AMPs and histones in the body, this would still be biologically relevant, but we wanted to understand whether the AMP/histone temperature sensitivity was indeed important. Thus, we performed the same assay using OH-CATH which is less temperature sensitive and found a 1.2-fold increase in mitochondrial damage (Extended Data Fig. 2c), which is comparable to the PI increase (1.2-fold) for OH-CATH at elevated temperature. We further quantified the increase of mitochondrial damage and PI uptake for LL-37 at 37°C vs. 40°C and found 1.7-fold increases for each. The change in AMP-induced membrane damage to *E. coli* is proportional to their effects on mitochondria, suggesting that changes in plasma membrane permeability are not a major cause of AMP uptakes. The damage on mitochondria appears to be largely due to the temperature-sensitive nature of AMP activity rather than a generic increase in AMP activity due to altered membrane permeability.

## Synergy reduces AMP-induced apoptosis

Since mitochondrial damage can trigger apoptosis^45,46^, we wondered whether the AMP- or AMP/histone-induced damage could lead to cellular apoptosis. To test this, we used apoptosis dye Annexin V which binds to phosphatidylserine that flips to the outer membrane leaflet during early apoptosis. We found that treating cells with moderate levels of external LL-37 at 37°C did not increase apoptosis, but that a switch to 40°C did, when the same LL-37 concentration was used (Fig. 3e). Further, as culturing the cells at 40°C did not increase apoptosis unless the LL-37 were added extracellularly, the presence of the AMPs appears to directly increase mitochondrial damage. Finally, when we combined AMP and histone, with concentrations chosen to have the same antibacterial efficacy as the AMPs alone, we found that cellular exposure to the dual treatment did much less mitochondrial damage than the AMP-alone treatment (Fig. 3e) —the untreated vs. dual treatment were statistically similar, in contrast to the LL-37 treatment, where apoptosis was very significantly increased at 40°C. This suggests that one advantage of the synergistic effects of LL-37 and histone over LL-37 alone is that the synergistic activity is specifically tailored to affect bacteria, therefore reducing collateral mitochondrial damage.

## Temperature reduces LD-mitochondrial contact

Interestingly, an increase in temperature alone (without LL-37 or histones) did not impair mitochondria (Fig. 3b, untreated). This was initially surprising to us, since even without exogenously supplying LL-37 and histones, both molecules are already present on lipid droplets (LDs)^37^, and we would expect LD-mitochondrial interactions to result in contact between mitochondria, LL-37 and histones, which would damage the mitochondria at the higher temperatures due to increased LL-37/histone activity. In our past work, we found that the body recognized infection and responded by increasing the concentration of histones and LL-37 on the LDs^23^. One of the corollaries of that increase was a concomitant decrease in LD-mitochondrial contacts^23^, presumably to protect the mitochondria. Indeed, in addition to our work here, past studies suggest that histones destabilize mitochondrial membranes^47^. Thus, we wondered whether the mitochondrial insensitivity to temperature could be due to the same mechanism—a decrease in LD-mitochondrial contact, achieved by increasing the LD-mitochondrial distance at higher temperatures.

**Fig. 4 |:**
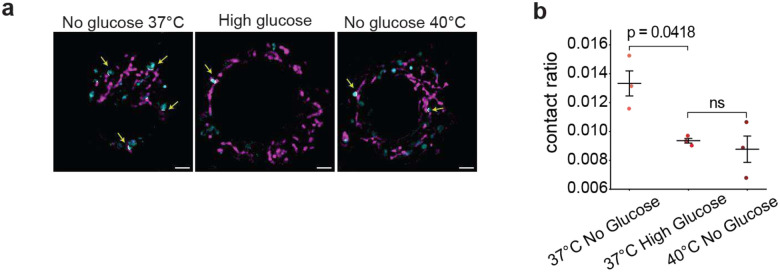
Lipid droplets move away from mitochondrial at febrile temperatures. **a**, Representative structured illumination microscopy (SIM) images of mitochondria (magenta) and lipid droplets (cyan). White lines indicate the contact regions. **b**, Quantification of lipid droplet-mitochondria contact area from three biological replicates. Black bars indicate mean and error bars represent SEM from three replicates. Scale bar = 2 μm. P-values greater than 0.05 are considered non-significant (ns).

In order to assess LD-mitochondrial contact in macrophages, we developed code to measure their distance in three dimensions. First, we measured contacts at 37°C under two different metabolic states: in a high-glucose state when cells were running off of glucose and LD-mitochondrial interactions were expected to be minimal^48^, and a low-glucose state when LDs were docked to mitochondria, so that they could be actively used to provide fatty acids for fatty acid metabolism. As expected, we found (Fig. 4a,b) that average LD-mitochondrial contacts were much more significant in the low-glucose state than in the high-glucose state. We next looked at LD-mitochondrial distances in a low-glucose state at 40°C, and found that LD-mitochondrial distances were much greater than at 37°C. In fact, they were comparable to the high-glucose condition at 37°C (Fig. 4a,b), suggesting minimal LD-mitochondrial interactions at 40°C under low glucose. Thus, it appears that at high temperatures, LDs move away from mitochondria, to avoid undue mitochondrial damage. As discussed below, this may explain why metabolism is known to be altered at higher temperatures.

## AMP damage is evolutionarily tuned

**Fig. 5 |:**
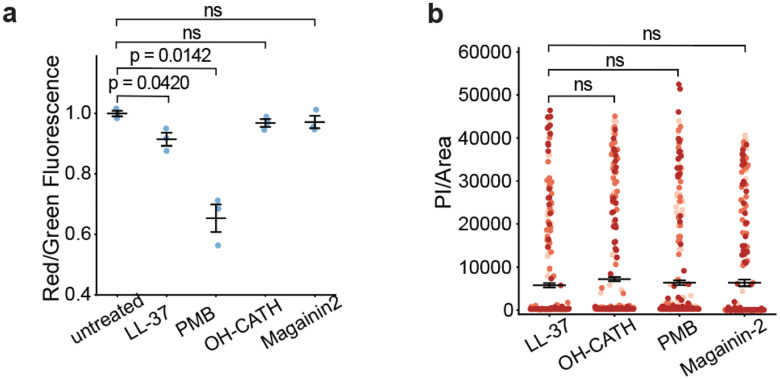
Cold-blooded animals AMPs are tuned to reduce mitochondrial damage. **a**, Quantification of mitochondrial activity of macrophages J774.1 using JC-1 red/green fluorescence ratio. Treatments were performed with 20 μg ml^−1^ LL-37, 3 μg ml^−1^ OH-CATH, 1 μg ml^−1^ PMB and 40 μM magainin-2 at 37°C for 1 hour. **b**, Quantification of PI per cell under the same treatment conditions. Black bars indicate mean and error bars represent the SEM from three replicates. P-values greater than 0.05 are denoted as non-significant (ns).

Our observations are consistent with the general hypothesis that the fever response is protective: it is being used to dramatically increase antibacterial killing activity when needed, while still protecting mitochondria via a combination of moderate AMP activity at body temperatures, together with increased separation of LD-anchored AMPs from mitochondria at high temperatures. However, it raises an interesting question: how do non-mammalian animals—which cannot raise their body temperature—have effective antimicrobial activity while limiting damage to mitochondria? We initially thought that perhaps AMPs and histones were stored somewhere other than LDs, to prevent mitochondrial exposure, but that seems unlikely since we found high levels of histones on Drosophila LDs^37^. Instead, given that AMP activity—at least as far as temperature—can be selectively tuned evolutionarily, we wondered if AMP activity might be further tuneable as far as selectivity. That is, might it be possible to tune AMPs so that they increase bacterial damage relative to mitochondrial damage? To test this idea, we first looked at PMB, a last-resort antibiotic which is an AMP that is produced by the bacterium *Bacillus polymyxa* to fight other bacteria, and presumably has no evolutionary pressure to avoid mitochondrial damage. When we chose a PMB concentration to match bacterial damage done by LL-37 (Fig. 5b), and then tested both PMB and LL-37 on cells to assess mitochondrial impact, we found that PMB did significantly more mitochondrial damage (Fig. 5a). This was our first suggestion that it indeed might be possible to evolutionarily tune selectivity to bacteria vs mitochondria. With this in mind, we next did the same experiment to compare LL-37 activity to OH-CATH (from the king cobra). We found that with similar antibacterial activity (Fig. 5b), OH-CATH did much less mitochondrial damage than LL-37 (Fig. 5a). Finally, we repeated the experiment with magainin-2. Here, because magainin is optimized to work at lower temperatures in frogs, to match the antimicrobial activity of the other AMPs at 37°C, we had to use a rather high dosage of magainin. Nonetheless, at this magainin dosage, its effect on mitochondria was quite low, comparable to that from OH-CATH, and significantly less than that due to LL-37. Thus, it appears that it is indeed possible to tune relative antibacterial vs anti-mitochondrial activity, and our data is consistent with the hypothesis that non-mammalian animals such as snakes and frogs have used this route to avoid mitochondrial damage. We did not include pelteobagrin in the above study because it is not active in the same medium as the others, so there was no clean way to do such a comparison.

## Discussion

The mechanisms that improve clearing of bacteria through fever have not been understood. Here, we have found that the antimicrobial activity of AMPs and histones increases significantly with temperature. At the heart of this mechanism is the histone/AMP synergism. The activity of both the LL-37 and histones is temperature dependent, and their synergistic combination achieves multiplicative antimicrobial activity and thus takes better advantage of small changes in temperature (Extended Data Fig. 1a), allowing for a more dramatic increase in bacterial killing efficacy compared to LL-37 alone. While the antimicrobial activity of LL-37 rises with temperature, there is a significant trade-off for the host: LL-37 significantly increases damage to the mitochondria (Fig. 3b). However, for a fixed level of antibacterial activity, the combination of histones and LL-37 has less mitochondrial effects than LL-37 alone (Fig. 3b,d, Extended Data Table 1). Synergy that arises from histones and LL-37 thus has the added benefit of increasing antimicrobial activity while mitigating mitochondrial damage. Furthermore, it may be harder for bacteria to evolve resistance to the multi-pronged attack^49,50^, as this likely requires independent evolution along multiple axes.

AMPs have the highest activity near the typical body temperature of the animal from which they come. For animals that are able to raise their body temperature, the maximal antimicrobial activity occurs at slightly elevated temperatures. This is not an accident, as the AMPs from animals unable to raise their body temperatures do not show this activity. Our data thus suggests that AMP activity reflects an evolutionary trade-off: because of their lipid droplet-associated nature^37^, AMP antimicrobial activity also damages mitochondria, thus for mammals AMP activity at the normal body temperature must be kept moderate. When invading microorganisms are detected, the body temperature is increased, maximizing AMP activity and activating synergy with histones.

Our findings show that the cell avoids excessive damage from LL-37 at elevated temperatures by moving LDs away from mitochondria (Fig. 4a–b). The protection of mitochondria through distancing has an unexpected impact on metabolism: as LDs move away from mitochondria, the latter’s access to fatty acids is restricted, and so glycolysis is the best option. This interpretation addresses an open issue: why does heat induce a shift towards glycolysis and away from why fatty acid metabolism^51–53^? While it has been suggested that the impairment is due to protein unfolding^54^, our results suggest this could be attributed to LD-mitochondrial distancing instead in order to avoid the damaging effects of AMPs.

For non-mammalian animals, our data suggests a different path: instead of tuning AMP activity to be strongly temperature sensitive, and then using the fever response to kill bacteria, non-mammalians likely used evolutionary tuning of AMP activity to decrease mitochondrial damage while maintaining ability to kill bacteria. One obvious prediction from our work is that metabolism of non-mammalian animals should be much less temperature sensitive. Overall advantages of the different strategies remain to be explored in future work, however, from a practical point of view, we suggest that for antibacterial drug development, it might be prudent to start with AMPs from non-mammalian animal sources, as they are likely to have less off-target effects when applied systematically, as compared to bacterially derived AMPs such as PMB, or mammalian AMPs that may be optimized for temperature dependence rather than decreased mitochondrial damage.

Our data may help rationalize the relatively recent observation that, on average, human body temperatures have decreased over the last hundred years^55,56^. Above, we suggested that the AMPs activity was likely the result of an evolutionary compromise between maximizing bacterial killing efficacy and minimizing mitochondrial damage. Over the last hundred years, as sanitary conditions have improved, our day-to-day exposure to bacterial infections has decreased. Thus, it may be possible to re-evaluate the trade-off, and accept slightly worse bacterial protection, in exchange for better mitochondrial health. The simplest way to achieve this seems to be to lower the body temperature, thus lowering the AMPs efficacy, and their associated damage to mitochondria. Indeed, our data (Extended Data Fig. 2a) suggests that even a few degrees decrease in temperature can materially decrease mitochondrial damage. Specifically, LL-37 treatment that decreases mitochondrial activity by 0.6-fold at 37°C has no effect at 35°C (Extended Data Fig. 2b).

Finally, our data suggests that inhibiting fever could impair the body’s efficacy in fighting bacterial infections. Antipyretic drugs are commonly used to reduce moderate fevers during infection, though this could have the unintended effect of reducing antimicrobial activity. Indeed, even moderate (38.5°C) temperature is associated with significantly improvement in bacterial killing (Fig 1 and Extended Data Fig. 1a-b and Extended Data Table 2). The extent to which these matters in a clinical setting remains to be explored more fully, but our data suggests that rather than decreasing body temperature, perhaps local heating at the sites of infection should be more actively employed. With the dramatic increase in antibiotic-resistant bacteria, a deeper understanding of how the body fights infection becomes increasingly important.

## Methods

### Bacterial Growth Conditions and Reagents

*E. coli* MG1655^57^ were streaked on petri dishes with LB-Miller (BD Biosciences, Franklin Lakes, NJ) and 2% Bacto agar (BD Biosciences). Plates were incubated at 37°C overnight to obtain single colonies. Single colonies were cultured into MinA+ medium (4.5 g KH_2_PO_4_, 10.5 g K_2_HPO_4_ 1 g (NH_4_)_2_SO_4_, and 0.5 g sodium citrate • 2H_2_O per 1 L water) supplemented with 1 mM MgSO_4_, 0.2% glucose and 0.1% casamino acids^27^. The cultures were grown overnight at 37°C on a roller drum at 18 rpm, then diluted 1:100 to fresh medium and grown until OD_600_ reached 0.2 to 0.4. Then exponential phase cells were diluted to an OD_600_ of 0.04 into fresh medium before treatment.

Antimicrobial agents calf thymus histones II-A (Sigma-Aldrich, St. Louis, MO), bovine histone H1 (Sigma-Aldrich), human cathelicidin LL-37 (Anaspec, Fremont, CA), polymyxin B sulfate salt (Sigma-Aldrich) were prepared in 0.1M sodium bicarbonate (pH 8.3) and stored at −20°C. OH-CATH (NovoPro Bioscience), Pelteobagrin (NovoPro Bioscience) and Magainin-2 (AnaSpec, Fremont, CA) were prepared in water and aliquots were stored at −80°C.

### Macrophage Growth Conditions

J774.1 mouse macrophages were cultured in T75 flasks with 10% fetal bovine serum (Cytiva HyClone, Fisher Scientific), 1% penicillin-streptomycin (Life Technologies) in DMEM base medium with high glucose (Gibco, Life Technologies) in 37°C incubator with 5% CO_2_. For no glucose treatment, cells were transferred to medium with 1% penicillin-streptomycin in DMEM base medium with no glucose (Gibco, Life Technologies) for 24 h before the experiment. At least three passages were done before the experiment.

### Epifluorescence Microscopy

We used a Nikon Eclipse Ti-E microscope (Nikon, Melville, NY) containing a 100X Plan Apo (1.45 N.A.) objective, a Sola light engine (Lumencor, Beaverton, OR), an LED-DA/FI/TX filter set (Semrock, Rochester, NY) containing a 409/493/596 dichroic and a Hamamatsu Orca Flash 4.0 V2 camera (Hamamatsu, Bridgewater, NJ). For propidium iodide (PI) fluorescence, *E. coli* was stained with 8 uM PI for 10 min and 10 μL of culture was added on slides with 1% agarose before imaging. 575/25 nm excitation and 641/75 nm emission filters were used.

For mitochondrial membrane potential, J774.1 mouse macrophages were plated into a 60×15 cm Falcon Petri dish with cover glass coated with 0.1% (w/v) poly-L-lysine (Sigma-Aldrich) on top 24 h before the experiment. Treatment was directly added into petri dish for 1 h. And JC-1 membrane potential detection kit (Biotium) was used to stain cells for 20 min at 37°C, washed twice with PBS, pH 7.4 (Thermo Fisher Scientific). Microchambers were made using cover glass attached to slides by double-sided tape and imaged immediately. We used the 575/25 nm excitation and 641/75 nm emission filters for the red channel and 474/27 nm excitation and 525/45 nm emission filters for the green channel. For apoptosis imaging, Annexin V Apoptosis Detection Kits (Thermo Fisher Scientific) were used to stain the cells for 15 min at 37°C, then washed twice with PBS and made microchamber before imaging. 575/25 nm excitation and 641/75 nm emission filters were used for imaging apoptosis. Images were obtained using Nikon NIS-Elements version 4.5 and analyzed using AVIassembleGUI^58^ version 1.2c in MATLAB R2017a (MathWorks, Natick, MA) (see ‘Code Availability’ section).

### Lattice SIM2 Microscopy

For mitochondrial and lipid droplets contact imaging, J774.1 macrophages were subcultured in 35 mm μ-Dish with ibiTreat (ibidi, Fitchburg, USA) 24 h before the experiment. The treatment was done for 1 h at 37°C or 40°C. Before imaging, stain with 100 nM of Mitotracker (Thermo Fisher Scientific) and 1 μg ml^−1^ BODIPY (Thermo Fisher Scientific) for 20 min and imaged immediately. Images were acquired using Elyra 7 Lattice SIM (Carl Zeiss, White Plains, NY) using a 63x/1.4 Oil Plan-Apo objective and dual camera: 2 pco.edge 4.2 camera system with a 0.329 μm-interspaced Z-stacks. Image was performed under 5% CO_2_ at 37°C or 40°C. 561 nm and 488 nm lasers were used for mitochondrial and lipid droplets respectively. We used Zen Black edition (3.0) to do super-resolution reconstruction with SIM2, 3D Leap mode, regularization weight of 0.065, and iteration 20. For contact area analysis, we developed an ImageJ (2.16.0/1.54p) Macro that can batch process two-channel 3D SIM images in a memory efficient way, by Gaussian blur, background subtraction, thresholding calculates contact area between two channels. The contact area is normalized by the total volume of lipid droplets and output as contact ratio (see ‘Code Availability’ section).

### CFU Assays

Overnight *E. coli* cells were grown to mid-exponential phase in MinA+ medium, 1:20 dilution into fresh medium then treated with antimicrobial peptides, histones or dual treatment for 1 h at indicated temperatures on block heaters and water baths. After treatment, cultures were diluted in ten-fold serial dilutions from 10^0^ to 10^6^ into fresh medium, 10 μl droplets were plated onto non-selective LB-Miller agar plates, incubated for 18 h at 37°C, and counted for single colonies.

### Statistical Analyses

Two-tailed t-tests with unequal variances for all data were performed with Python 3.10.9 using scipy.stats.

### Data and Materials Availability.

All data needed to evaluate the conclusions in the paper are present in the paper and/or the Supplementary Information. Raw data used for statistical analyses are available in Source Data. All other raw data used in this study is available upon request. Correspondence and requests for materials should be addressed to asirya@uci.edu. The custom analysis code is available on Github at https://github.com/asirya/MitoContactArea

## Supplementary Files

This is a list of supplementary files associated with this preprint. Click to download.

- ExtendedData.pdf

Supplementary Information is available for this paper.
